# The Functional Classification and Field Test Performance in Wheelchair Basketball Players

**DOI:** 10.1515/hukin-2015-0050

**Published:** 2015-07-10

**Authors:** Susana María Gil, Javier Yanci, Montserrat Otero, Jurgi Olasagasti, Aduna Badiola, Iraia Bidaurrazaga-Letona, Aitor Iturricastillo, Cristina Granados

**Affiliations:** 1 Department of Physiology. Faculty of Medicine and Dentistry. University of the Basque Country (UPV/EHU).; 2 Department of Physical Education and Sport. Faculty of Physical Activity and Sport Sciences. University of the Basque Country (UPV/EHU).; 3 Adapted Sport Federation of Gipuzkoa, Donostia-San Sebastian, Spain.

**Keywords:** disability, spinal cord injury, speed, agility, endurance, strength

## Abstract

Wheelchair basketball players are classified in four classes based on the International Wheelchair Basketball Federation (IWBF) system of competition. Thus, the aim of the study was to ascertain if the IWBF classification, the type of injury and the wheelchair experience were related to different performance field-based tests. Thirteen basketball players undertook anthropometric measurements and performance tests (hand dynamometry, 5 m and 20 m sprints, 5 m and 20 m sprints with a ball, a T-test, a Pick-up test, a modified 10 m Yo-Yo intermittent recovery test, a maximal pass and a medicine ball throw). The IWBF class was correlated (p<0.05) to the hand dynamometry (r= 0.84), the maximal pass (r=0.67) and the medicine ball throw (r= 0.67). Whereas the years of dependence on the wheelchair were correlated to the velocity (p<0.01): 5 m (r= −0.80) and 20 m (r= −0.77) and agility tests (r= −0.77, p<0.01). Also, the 20 m sprint with a ball (r= 0.68) and the T-test (r= −0.57) correlated (p<0.05) with the experience in playing wheelchair basketball. Therefore, in this team the correlations of the performance variables differed when they were related to the disability class, the years of dependence on the wheelchair and the experience in playing wheelchair basketball. These results should be taken into account by the technical staff and coaches of the teams when assessing performance of wheelchair basketball players.

## Introduction

Wheelchair basketball players must have an optimal speed, agility, strength, power, endurance, technical and tactical skills to display good performance during the games. However, these athletes may have been affected by a wide range of injuries and diseases; thereby, they encompass different levels of disability leading to considerable differences in the capacity to perform. In order to balance the large variety in the functional capabilities of the players and to ensure that all eligible players have an equal right and opportunity to play, the International Wheelchair Basketball Federation (IWBF) designed a classification system based on the player’s physical capacity to execute fundamental basketball movements; pushing the wheelchair, dribbling, shooting, passing and catching, rebounding and reacting to contact (IWBF, 2010).

Thus, this classification reflects the degree of the disability of athletes, and as such, it is a central aspect in wheelchair sports (Goosey-Tolfrey and Leicht, 2013). Consequently it is interesting to know if the aforementioned classification also reflects the functional performance of the wheelchair athletes while they are practicing their sport. In this respect, to our knowledge, only a few studies have attempted to identify the relationship between the functional IWBF classification and performance. Moreover, most of these studies were laboratory-based tests and they analysed incremental cardiopulmonary exercise testing to measure the ventilatory threshold, peak oxygen uptake, and the Wingate test to measure anaerobic power and capacity (De Lira et al., 2010; Molik et al., 2010a; Morgulek-Adamowiccz et al., 2011). Laboratory tests allow the development of physical tests on strictly monitored participants and under particularly well controlled external conditions. On the contrary, field-based tests are easier to execute and interpret; and they also mimic more closely the actions and the movements (i.e. side cutting manoeuvres) of the training sessions and games, representing performance of the athletes in a more exact way.

Since the classification of the IWBF reflects the functional capacity of disabled athletes, we hypothesized that there would be a correlation between the different levels of the classification and the performance in a wide range of field-based tests. Thus, the aim of the present study was to ascertain if the IWBF classification, the type of injury (spinal cord injury vs. non-spinal cord injury) and the wheelchair experience (both for training and everyday activities) were related to performance in short- (strength, power, agility, speed and technical skills) and long-duration (endurance) field tests.

## Material and Methods

### Participants

Thirteen male wheelchair basketball players, belonging to the Spanish national WB third division league participated in this study. All of them conducted two training sessions and played one game per week. Written informed consent was received from all players after verbal and written explanation of the experimental design and potential risks of the study were presented. The Ethics committee of the University of Basque Country for Research on Human Subjects approved the study. The measurements were performed according to the ethical standards of the Helsinki Declaration.

### Classification

In order to define the disability of each player, the International Wheelchair Basketball Federation (IWBF) designed a classification system based on the player’s physical capacity to execute fundamental basketball movements; pushing the wheelchair, dribbling, shooting, passing and catching, rebounding and reacting to contact. In this sense, the classification of the players is made to describe different variables such as the volume of action (the limit to which a player can move voluntarily in the vertical plane, the forward plane and the sideways plane), the sitting position and the pelvic stability (IWBF, 2010). Thus, players are grouped into categories (classes) from 1.0 (being the player with least physical function) to 4.5 (being the player with most physical function). This classification is the players’ “playing points” and at any given time in a game the five players on court must not exceed a total of 14 playing points (IWBF, 2010).

In order to be allowed to play each player must pass a medical examination to determine his or her class. This examination is undertaken by the classifiers. There are three levels of classifiers in wheelchair basketball: national, regional and international. Briefly, these classifiers must be involved in wheelchair basketball and they must attend a classification course for the purpose of training classifiers. Classification is based on the function of the trunk, the upper extremities, the lower extremities and the hands. Thus, it relies on the movement and the stability of the trunk of the player.

In the present study, the participants were classified according to the Classification Committee of the IWBF as: Class 1 (n=1), class 1.5 (n=1), class 2 (n=3), class 2.5 (n=1), class 3 (n=2), class 3.5 (n=2), class 4 (n=2) and class 4.5 (n=1).

### Measures

#### Anthropometric measurements

Height and sitting height (cm) were measured to the nearest 0.1 cm using a stadiometer (Holtain Ltd^®^, Crymych, United Kingdom). Body mass was obtained to the nearest 0.1 kg using an electronic scale (Seca Instruments Ltd^®^, Hamburg, Germany). Sitting height and body mass were measured as described by Vanlandewijck et al. (2011). Skinfold thicknesses (measured in mm) were measured at four sites (triceps, subscapular, abdominal and suprailiac) using a skinfold caliper (Harpenden, England) and the sum of these four measurements was calculated (sum of skinfolds). The perimeter of the relaxed arm and isometrically contracted arm (90º flexion) were measured using a tape measure (Seca Instruments Ltd^®^, Hamburg, Germany). All measurements were taken following the guidelines outlined by the ISAK (International Society for the Advancement of Kinanthropometry) by the same researcher.

#### 

##### Speed

**Figure 1:**
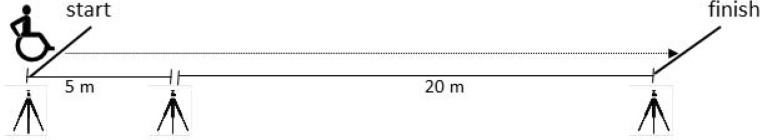
Speed test

The 20 m sprint with and without a ball (Figure 1): In the sports hall the basketball players performed a 20 m flat sprint test. Also, they performed a similar test with a ball, adhering to the IWBF rules for dribbling (De Groot et al., 2012). The coefficients of variation for the 20 m sprint with and without a ball were 1.41% and 3.03%, respectively.

In both, test performance was measured using electronic timing lights (Polifemo, Microgate, Bolzano, Italy) positioned at 5 m and 20 m and placed 0.4 m above the ground with accuracy of ±0.001 s. The starting position of the players was 0.5 m before the first timing light. All the tests were performed three times with 2 min of recovery in between. The best result of each test was used for further analysis.

##### Agility

**Figure 2:**
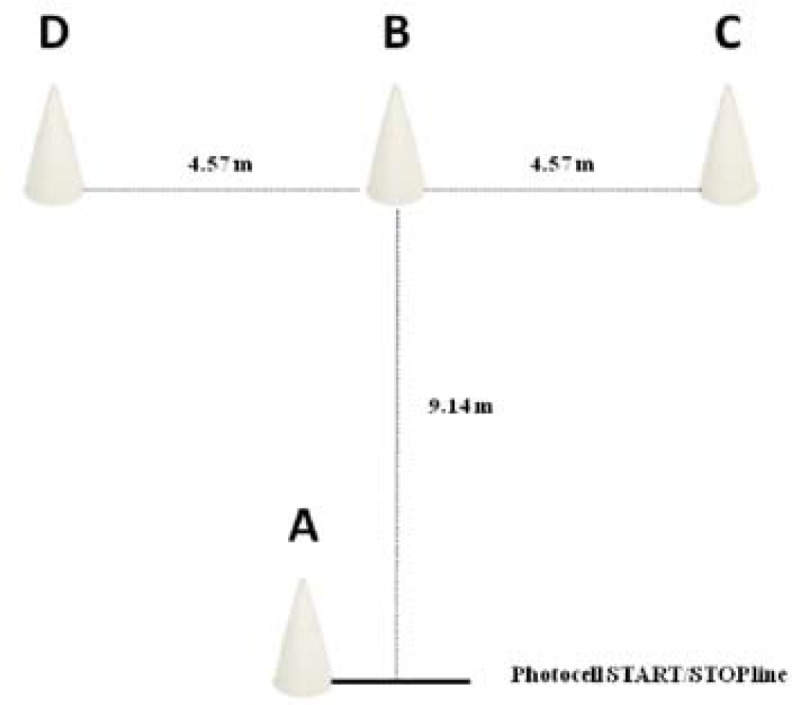
Agility T-test

T-test: The participants began with the wheels 0.5 m from cone A, and completed the circuit as follows (Figure 2) using the protocol by Sassi et al. (2009), modified to perform with a wheelchair and always using forward movements (Yanci et al., 2015). A–B displacement (9.14 m): At his/her own discretion, each subject moved quickly forward to cone B and touched the top with the right hand. B–C displacement (4.57 m): Facing forward they moved to the left to cone D and touched the top with the left hand. C–D displacement (9.14 m): The participants then moved to the right to cone D and touched the top. D–B displacement (4.57 m): They moved back to the left to cone B and touched the top. B-A displacement (9.14 m): Finally, the participants moved as quickly as possible and returned to line A. All participants performed the test 3 times with at least 3 min rest between trials. The total distance covered was 36.56 m and the height of the cones was 0.3 m. Seven days later, the retest was performed under the same conditions. A photocell (Migrogate Polifemo Radio Light®, Bolzano, Italy) located over cone A was used to record the time. Time measurement started and finished when the subject crossed the line between the tripods. The calculated margin of error was ±0.001 s and the sensors were set approximately 0.40 m above the floor. The coefficient of variation was 2.58%.

**Figure 3:**
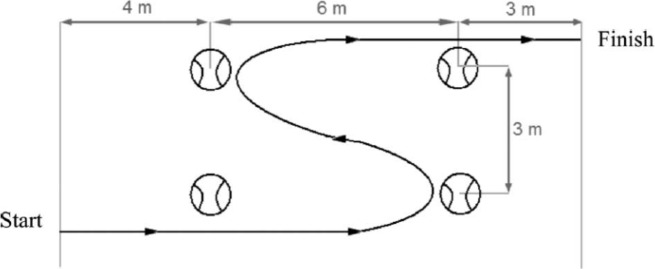
Pick-up test (De Groot et al., 2012)

Pick-up the ball: From a stationary position the participant had to start propelling and pick up four basketball balls from the floor as previously described by De Groot et al. (2012) (Figure 3). The total time taken to complete the test was recorded with a photocell (Migrogate Polifemo Radio Light^®^, Bolzano, Italy) located over the start and finish lines. All participants performed the test 3 times with at least 3 min rest between trials. The coefficient of variation for this test was 6.61%.

##### Strength and power

Hand dynamometry test: To measure the strength of the upper extremities, basketball players performed a handgrip test. They squeezed the dynamometer (Jamar, USA) with a maximum isometric effort for 5 s with a rest period of at least 60 s and the highest value was used to determine maximal grip strength (kg).

Maximal pass: As described by De Groot et al. (2012), the participant began in the middle of the baseline, front wheels behind the line, and passed a basketball as far as possible from a stationary position. The distance between the participant and the spot where the ball hit the floor was measured (in meters). The final score was the average distance of five passes.

**Figure 4:**
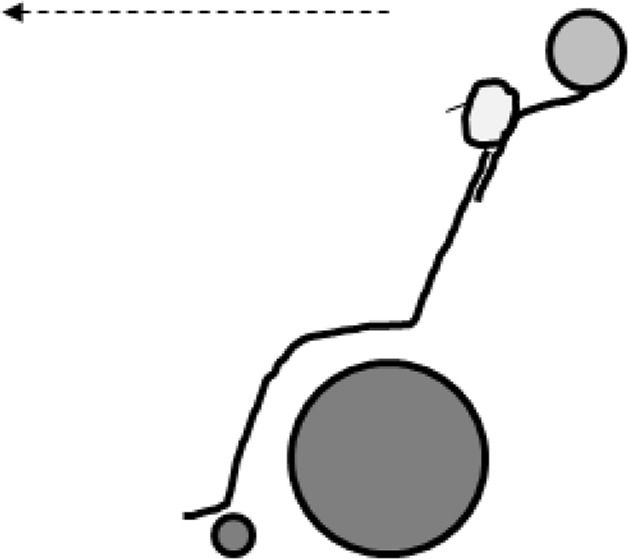
Medicine ball throw

Medicine ball: Using a similar position to the maximal pass, participants had to throw a 5 kg medicine ball as far as possible (Gonaus and Muller, 2012) (Figure 4). The distance was measured in meters. Each participant made three attempts and the best was used for further analysis.

##### Endurance

**Figure 5:**
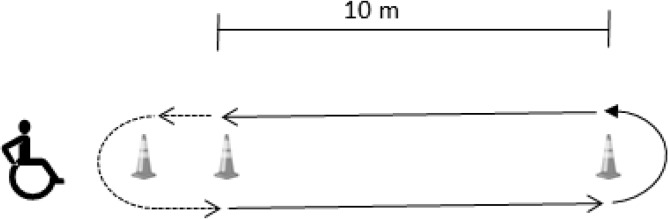
Endurance test

Yo-Yo intermittent recovery adapted test (Yo-yo ITa): Level 1 version of the Yo-Yo test was completed according to previously described methods (Castagna et al., 2008). Due to the differences between running and propelling the wheelchair, the distance covered in the shuttle run was reduced to 10 m (Yanci et al., 2015) (Figure 5). The total distance covered during the test was measured (meters). The heart rate (HR) was recorded at 5 s intervals by telemetry (Polar Team Sport System^®^, Polar Electro Oy, Finland) during the entire test. Within a week, participants repeated this tests and the ICC for the distance in the test was 0.94.

Before (pre-test) and immediately after the endurance test (post-test) earlobe capillary blood-samples were obtained for the determination of lactate concentrations (Lactate Pro LT-1710^®^, ArkRay Inc Ltd, Kyoto, Japan).

### Experimental Design

The tests were performed on a synthetic indoor court in the beginning of the season. The players were instructed to perform all tests at maximum intensity. Players did not perform strenuous exercise within 48 h prior to testing.

Testing was conducted over two different sessions separated by one week. During the first testing session anthropometric measurements, the handgrip, sprint and agility tests were performed; and during the second one, the strength and endurance performance were evaluated. Before each testing session a standardized warm-up was undertaken by the participants.

Participants age (years), type of injury: spinal cord injury (SCI) and non-SCI, IWBF class, the use of the wheelchair for everyday activities (in years) and experience in wheelchair basketball (in years) were recorded.

### Statistical analysis

Mean ± standard deviation and coefficient intervals at 95% (CI 95%) were used to describe the sample of participants. Skinfold thickness and the results of the Pick-up test were transformed into logarithms. Normality of the data was proved using the Kolmogorov-Smirnov and Saphiro-Wilk tests to verify the need of parametric or non-parametric tests to be applied. To analyse the correlation between different variables Pearson’s correlations or Spearman’s Rank Order were performed. The median value of the IWBF classification was calculated and two groups of players were created: players with a class above the median and players below the median. Also, participants were divided according to their injury into players with a spinal cord injury (SCI) and players with other type of injuries (non-SCI). To identify significant differences in the variables among two groups of players a Mann-Whitney U test was performed. To measure the effect size, Cohen’s d was evaluated. Threshold values for effect size statistics were 0.2, 0.5 and 0.8 for small, medium and large effect sizes, respectively (Cohen, 1988).

Statistical analyses of data were performed using the Statistical Package for the Social Sciences 17.0 software package (SPSS). The level of significance was set at *p*<0.05.

## Results

The descriptive statistics of the participants are shown in Table 1.

The class of the participants positively correlated (*p*= 0.01–0.05) with the body mass (r= 0.68), height (r= 0.77), contracted arm perimeter (r= 0.68), hand dynamometry (r= 0.84), maximal pass test (r= 0.67) and the medicine ball throw (r= 0.67) in both the total group and also the SCI players (Table 2). However, there was a statistically significant negative correlation (*p*≤0.01) only in the SCI players between the IWBF classification and the 5 m sprint test (r= −0,92), the 20 m sprint test (r= −0,92), the 5 m with a ball (r= −0,89), the 20 m with a ball (r= −0,82), the T-test (r= −0,81) and also the distance covered in the modified Yo-yo ITa test (r= 0,82). Meaning that the players with a higher class in the classification of the disability performed better.

The years of experience using a wheelchair were negatively correlated (*p*≤0.01) with the 5 and 20 m sprint test as well as the T-test, but only when the whole group was taken into account. Thus, players who used the wheelchair for everyday activities performed better in the aforementioned tests. On the other hand, the experience in basketball playing only correlated negatively (*p*≤0.05) with the 20 m test with a ball (r= −0.68) and the T-test (r= −0.57).

Players with a disability class below the median value had more experience (*p*≤0.05) using a wheelchair (d= 1.41) and playing wheelchair basketball (d= 0.92). They also had lower body height (*p*≤0.05, d= 2.01), body mass (*p*≤0.05, d= 1.64) and smaller arm perimeter (*p*≤0.05) in both, the relaxed (d= 1.49) and the contracted (d= 1.22) positions. Moreover, players with the class above the median value displayed a larger hand-grip strength (*p*≤0.05, d= 2.06), maximal pass (*p*≤0.05, d= 2.05) and medicine ball throw (*p*<0.05, d= 1.43) (Table 3).

On the other hand, players with a SCI had lower body height (d= 2.33) and mass (d= 1.56). They also had a lower hand-grip strength (d= 1.49), maximal pass (d= 1.04) and medicine ball throw (d= 0.83).

In contrast, these players performed better in the following tests: 5 m sprint (d= 1.55), 20 m sprint (d= 1.33), 5 m with a ball (d= 1.02), 20 m with a ball (d= 0.95), the T-test (p≤0.05, d= 2.00) and the pick-up test (d= 1.16). Moreover, athletes with SCI covered a longer distance in the endurance test (d= 1.86).

## Discussion

In the present study we analysed the relationship among the IWBF classification together with the use of the wheelchair and a variety of field-based tests designed to measure speed, agility, technique, strength, power and endurance performance in a team of wheelchair male basketball players. We observed that the correlations between performance variables differed when they were related to the disability class, the years of use of the wheelchair and the experience in playing wheelchair basketball.

The parameters that mainly rely on strength and power, such as the hand dynamometry and the tests measuring throwing ability (maximal pass and the medicine ball throw) were positively related to the disability class. Likewise, participants with a higher class (class above the median) displayed the best performances (statistically significant differences and large effect sizes). The player’s classification system is based mainly on the competence of a classifier to recognize a player’s physical ability in executing fundamental movements in wheelchair basketball. Classifiers are trained to observe and analyse trunk movement during the execution of basketball skills, such as pushing and handling the wheelchair, dribbling and passing, shooting and rebounding the ball (Vanlandewijck et al., 2004). Meanwhile, trunk strength and pelvic stability are key elements in the movements of throwing and passing a ball particularly from a sitting position. In the throws, the less disabled athletes are able to use their greater functional muscle mass to accelerate the implement during the force-producing phase of the throw which translates into greater acceleration of the object, a greater release velocity, thus a greater throwing distance (Higgs et al., 1990). Moreover, different biomechanics have been observed during free throw shots in basketball players of higher *vs*. lower classes (Goosey-Tolfrey et al., 2002; Malone et al., 2002), thus, players from different IWBF classes tend to rely on different kinematic strategies to produce successful release conditions. Thereby, participants with strongest trunk muscles and best pelvic stability, like the players with higher IWBF class in our study, were able to throw the ball the furthest. Similarly, large differences were observed in the athletes of the highest and the lowest (old athletic) classes in the throwing events (discus, shot and javelin) in the International Stoke Mandeville Games in 1987. These differences were more evident than the differences in the track events (Higgs et al., 1990).

On the other hand, the maximal isometric strength exerted by the forearm muscles in humans during a hand dynamometry test is proportional to their size whatever the age (Tonson et al., 2010). In the present study the perimeter of the forearm was not measured, but the perimeter of the upper arm both in the relaxed and flexed position was smaller in the participants with the lowest class (below the median) and presumably also the diameter of the forearm; thus preventing them to produce a stronger prehensile force. For the same reason, players with SCI displayed lower handgrip strength than the nonmedullar participants, with a difference not statistically significant but with a large effect size (d= 1.49).

In our study, when the whole team was taken into account, the velocity measured in the 5 m and 20 m sprint, the 20 m with a ball and the agility T-test were correlated (negatively) to the use of the wheelchair in years, but not with the IWBF classification class. In contrast, in other studies it has been observed that anaerobic power is greater in wheelchair basketball participants of a higher IWBF class compared to players of lower classes (i.e. more disabled) measured with different protocols of arm-cranking exercise (De Lira et al., 2010; Molik et al., 2010a). However, there are significant biomechanical and physiological differences between an arm cranking exercise (or handcycle) and wheelchair exercise (Dallmeijer et al., 2002). Moreover, wheelchair dependent participants have distinct biomechanics (propulsion) and energy cost (efficiency) than non-accustomed able-bodied participants and even able-bodied participants trained for three weeks (Croft et al., 2013). Also, wheelchair-dependent people have higher mechanical efficiency than non-accustomed able-bodied participants during manual wheelchair ergometry (Brown et al., 1990). Thus, it is not surprising that in our study players with SCI, that use their wheelchair for everyday activities, performed better in the velocity and agility tests (T-test and Pick-up test) compared to the players that only use the wheelchair for training and playing the games.

To our knowledge only few studies have aimed to determine the relationship between the functional classification level and sport specific skill tests, for instance wheelchair basketball (De Groot et al., 2012; Hutzler, 1993; Molik et al., 2010b) and wheelchair rugby (Morgulek-Adamowicz et al., 2011). Similarly to our study, De Groot et al. (2012) did not find any differences between the players of a low (<4) and high (>4) IWBF classification level in short-term performance field-based tests. While the rest of the authors observed that the participants with a higher classification class performed better; these differences were particularly significant amongst the participants of both ends of the classification. The difference with our study may come from the group of participants. In our study players belonged to one team, they trained and played together and in the same conditions. Whereas the participants of Molik et al.’s study (2010b) where more heterogeneous, and belonged to different teams; additionally they included other different pathologies such as cerebral palsy. On the other hand, Hutzler (1993) observed a moderate correlation (r= −0.64, p=0.031) between the class and 428 m racing trails (lasting for about 2–3 min), but not the 6 min test nor the slalom test.

In the present study the tests that required the best agility and technical aspect (T-test and 20 m with a ball) were related to the years of experience in playing basketball. Thus, not only everyday use of the wheelchair but also training seems to be important to have a good performance in the most complex and technically difficult tests (Vanlandewijck et al., 2011). It has been observed that even seven weeks of a low intensity training program can improve peak aerobic and sprint power output, efficiency and physical strain in untrained able-bodied male individuals (Van den Berg et al., 2010), demonstrating the importance of training particular skills to excel in wheelchair basketball.

Besides, a better propulsion technique, mechanical efficiency and agility to turn may be the cause for the SCI players to outperform the non-SCI players in the endurance test which is important due to the fact that 64% of the game consists of propulsion activation and 36% of the time braking activity (Coutts, 1992).

One of the limitations of our study is the small number of participants, particularly the players with non-SCI. However it is worth noticing that all of the players belonged to the same competitive team and therefore they trained and played together and the results may not be related to different training status. In any case, it would be very interesting to repeat the study in different teams of wheelchair basketball of the same level to reassure the results.

In summary, this set of easy-to-implement performance tests may be used by coaches. However, caution should be taken when relating the results to the disability because the relationship between the IWBF class and performance may vary, and we could only confirm our hypothesis partially. In fact, in this team of wheelchair basketball players, significant correlations were observed between the IWBF classification class and the power and strength of the arms and the body. In contrast, velocity and agility were related to the years of wheelchair use, whereas agility and technique were related to the years of wheelchair basketball training.

## Figures and Tables

**Table 1:** Descriptive statistics of the anthropometry and physical tests of the participants

	**Mean ± SD**	**C.I. 95%**	
Age (years)	33.30 ± 8.01	28.46	38.15
Use of the WC (years)^a^	10.30 ± 10.15	4.17	16.44
Playing experience (years)	5.86 ± 4.40	3.20	8.52
Sitting body height (cm)	86.80 ± 6.67	80.83	89.80
Body mass (kg)	75.80 ± 20.82	58.34	84.48
Σ skinfolds (mm)	71.55 ± 28.20	52.45	85.88
Relaxed arm perimeter (cm)	33.08 ± 3.32	30.96	35.92
Contracted arm perimeter (cm)	36.42 ± 3.29	34.33	38.51
Hand dynamometry (kg)	44.96 ± 9.98	38.57	51.26
Maximal pass (m)	9.15 ± 1.72	7.99	10.30
Medicine ball throw (m)	3.78 ± 0.66	3.33	4.32
5 m sprint (s)	1.86 ± 0.22	1.71	2.02
20 m sprint (s)	5.65 ± 0.45	5.34	5.95
5 m sprint with a ball (s)	2.09 ± 0.32	1.88	2.31
20 m sprint with a ball (s)	6.56 ± 0.66	6.11	7.01
T-test (s)	16.94 ± 1.23	16.11	17.77
Pick-up test (s)	16.37 ± 2.69	14.30	18.43
Lactate pre (mmol·l^−1^)	1.04 ± 0.41	0.77	1.30
Lactate post (mmol·l^−1^)	7.53 ± 2.5	5.93	9.12
HRmax (bpm)	173.5 ± 21.18	160.03	186.96
Distance (m)	1028.75 ± 399.21	775.11	1282.39

WC: wheelchair; Σ skinfold (mm): tricipital + subscapular + abdominal + suprailiac skinfolds. HRmax: maximum heart rate (beats per minute).

The lactate values, heart rate and distance refer to the endurance test.

SD: standard deviation, C.I.: coefficient intervals

**Table 2:** Correlations amongst an IWBF class, wheelchair use for everyday activities (number of years), and anthropometric and performance parameters. Values are shown for the total group and the spinal cord injury (SCI) sub-group

		IWBF Classification	Wheelchair use
Body mass	Total	0.68^*^	−0.85^***^
	SCI	0.25	−0.75^**^
Height	Total	0.77^**^	−0.84^**^
	SCI	0.68^*^	−0.75^*^
Σ skinfold	Total	0.48	−0.28
	SCI	0.45	−0.16
Contracted arm perimeter	Total	0.68^*^	−0.33
	SCI	0.85^**^	−0.15
Hand dynamometry	Total	0.84^*^	−0.36
	SCI	0.77^**^	−0.42
Maximal pass	Total	0.67^*^	−0.30
	SCI	0.88^**^	0.14
Medicine ball throw	Total	0.67^*^	−0.24
	SCI	0.86^**^	0.23
5 m sprint	Total	0.11	−0.80^**^
	SCI	−0.92^**^	−0.54
20 m sprint	Total	0.02	−0.77^**^
	SCI	−0.92^**^	−0.54
5 m sprint with a ball	Total	−0.07	−0.53
	SCI	−0.89^**^	−0.49
20 m sprint with a ball	Total	0.02	−0.65^*^
	SCI	−0.82^*^	−0.41
T-test	Total	0.33	−0.78^**^
	SCI	−0.81^**^	−0.45
Pick-up test	Total	0.51	−0.20
	SCI	0.09	−0.46
Lactate pre	Total	0.10	−0.24
	SCI	0.76	−0.39
Lactate post	Total	0.07	−0.56
	SCI	0.34	−0.60
HRmax	Total	0.20	0.33
	SCI	0.54	0.41
Distance	Total	0.04	0.36
	SCI	0.82^**^	−0.20

IWBF = International wheelchair basketball federation; Σ skinfold (mm): tricipital + subscapular + abdominal + suprailiac skinfolds. HRmax: maximum heart rate (beats per minute).

HRmax: maximum heart rate. The lactate values, heart rate and distance refer to the endurance test.

* p<0.05,

** p<0.01,

*** p<0.001

**Table 3:** Descriptive statistics of anthropometry and physical tests of the participants grouped according to the level of disability and the type of injury

	**IWBF class^a^**			**SCI injury**		
	**Below median**	**Above median**	**d**	**Non-SCI**	**SCI**	**d**
Age (years)	32.66±8.75	33.37±7.52	−0.08	33.80±8.92	32.66±7.56	0.13
WC use (years)	16.33±7.99	4.5±8.75^*^	1.41	-	14.88±8.82	-
Playing experience (years)	8.16±4.49	4.40±5.54^*^	0.92	4.05±3.67	7.11±4.37	−0.75
Sitting body height (cm)	82.06±3.37	91.53±5.74^*^	−2.01	94.20±1.70	84.33±5.76	2.33
Body mass (kg)	62.46±7.27	89.13±21.79^*^	−1.64	94.46±17.67	68.91±17.45	1.56
Σ skinfold (mm)	61.10±16.70	82.01±34.76	−0.76	86.86±42.20	66.45±23.05	0.65
Arm perimeter (cm)	31.06±1.41	35.10±3.55^*^	−1.49	34.36±3.09	32.65±3.46	0.51
Contracted arm (cm)	34.66±2.02	38.18±3.51	−1.22	37.10±3.63	36.20±3.37	0.25
Hand dynamometry (kg)	34.56±5.03	42.05±1.91^*^	−2.06	46.66±2.88	37.75±7.93	1.49
Maximal pass (m)	8.01±0.88	10.51±1.48^*^	−2.05	10.18±1.49	8.55±1.64	1.04
Medicine ball throw (m)	3.42±0.58	4.21±0.52^*^	−1.43	4.11±0.54	3.59±0.69	0.83
5 m sprint (s)	1.87±0.14	1.85±0.29	0.08	2.03±0.11	1.76±0.22	1.55
20 m sprint (s)	5.62±0.45	5.67±0.48	−0.10	5.96±0.26	5.47±0.45	1.33
5 m sprint with a ball (s)	2.10±0.18	2.09±0.42	0.03	2.29±0.33	1.98±0.27	1.02
20 m sprint with a ball (s)	6.46±0.53	6.64±0.79	−0.26	6.96±0.79	6.33±0.50	0.95
T-test (s)	16.51±1.02	17.29±1.37	−0.64	18.07±0.86	16.29±0.91^*^	2.00
Pick-up test (s)	16.04±3.14	16.63±2.61	−0.20	18.20±2.10	15.45±2.60	1.16
Lactate pre (mmol·l^−1^)	1.06±0.23	1.01±0.57	0.11	1.16±0.76	1.00±0.29	0.29
Lactate post (mmol·l^−1^)	7.58±3.21	7.48±1.88	0.03	6.33±2.00	7.93±2.63	0.68
HRmax (bpm)	169.66±25.63	177±15.57	−0.35	168.66±16.86	175.11±23.11	0.31
Distance (m)	980.83±330.7	1076.66±458.53	−0.23	646.66±64.29	1156.11±380.9^*^	−1.86

WC: wheelchair; Σ skinfold (mm): tricipital + subscapular + abdominal + suprailiac skinfolds.

HR: heart rate (beats per minute).

* p<0.05 Mann-Whitney U test.

The lactate values, heart rate and distance refer to the endurance test. d: Cohen’s d

a median value was 3
